# Utilizing mobile health and community informants to collect real-time health care data in extremely low resource environments

**DOI:** 10.7189/jogh.10.020411

**Published:** 2020-12

**Authors:** Peggy J Ebner, Natalie M Friedricks, Luso Chilenga, Ted Bandawe, George Tolomiczenko, Jared M Alswang, William B Belshe, Neeraj Sood

**Affiliations:** 1 Keck School of Medicine of the University of Southern California, Los Angeles, California, USA; 2 Chitipa District Health Office, Chitipa, Malawi; 3 University of Southern California, Los Angeles, California, USA; 4 Harvard Medical School, Boston, Massachusetts, USA; 5 David Geffen School of Medicine of the University of California, Los Angeles, California, USA; 6 Price School of Public Policy, University of Southern California, Los Angeles, California, USA

## Abstract

**Background:**

Mobile health provides promising opportunities to perform population surveillance in rural, impoverished, or unstable communities. The objective of this study was to test the efficacy and accuracy of data collected by community informants in extreme low-resource environments using electronic surveys and mobile phones.

**Methods:**

We carried out a population-based, cross-sectional survey between October and November 2017 measuring access to health care and prenatal services for pregnant women in the Northern Region of Malawi. The survey was conducted by members of the community who received one day of training and volunteered to conduct a survey for each live birth that occurred within their predetermined catchment area. A study member audited less than 2% of survey responses, where community informant responses were compared to community member self-reports.

**Results:**

A total of 915 survey responses were recorded by 21 community informants. These surveys recorded 621 live births and 4 cases of maternal mortality. This represents a maternal mortality rate of 0.64% (95% confidence interval (CI) = 0.2% to 1.6%), roughly equal to the United Nations Children’s Fund (UNICEF) estimate from 2015 of 634 per 100 000 live births, or 0.63%. This survey captured 120 births by adolescent mothers aged 15-19 out of 673 responses about maternal age. This represents 17.8% (95% CI = 15.1% to 20.9%) of all births, slightly higher than the UNICEF estimate of 143 per 1000 live births (14.3%). Finally, 51.7% of women were recorded as attending 4 antenatal care visits (95% CI = 47.8% to 55.7%), consistent with the 2015-2016 Demographic and Health Survey (DHS) value of 51%.

**Conclusions:**

The use of cellular phones and electronic surveys by community informants allowed for the real-time capture of data in an area where access is limited by seasonally impassable roads and unreliable cell reception. The data recorded by the surveys is comparable to accepted statistics in several measures. Community reporting of health care data can provide an efficient method of monitoring extremely rural or hard to reach communities.

Health surveillance and early detection of epidemiologic trends are crucial to maintaining a population’s health, but often it is difficult to effectively collect data in areas where infrastructure is poor [1-4]. Established methods of surveying populations, like the Demographic and Health Survey (DHS), are generally conducted every 5 years and survey large populations. While they provide crucial data about developing nations, they may fail to provide specifics about subgroups and cannot capture shorter term changes that occur between survey periods or pertain to the spread of infectious disease. Due to the increasing availability of mobile phones and cellular networks, it has been suggested that mobile health, the use of mobile devices to support the practice of medicine or population health, could provide efficient solutions to the growing demands of population health surveillance [5,6]. This study sought to assess the feasibility of using local community members to collect surveillance data in extremely rural and hard to reach areas using a pilot survey in Northern Malawi.

Malawi is one of the poorest countries in the world according to the International Monetary Fund, and is the recipient of significant aid to address the disproportionately high rates of maternal mortality and death of children under 5 [7,8]. As such, maternal health access and mortality rates represent well-studied measures of health access in an otherwise young and well population that facilitate a comparison between this study design and established methods.

Within Malawi, the Northern Region is less densely populated, contains more mountainous terrain, and is home to a segment of the population with less access to education and employment [9]. This region experiences frequent power outages (often in excess of 8 hours per day) and seasonal rains that make travel difficult and isolate areas within the region for months at a time [10]. Despite these geographic challenges, inhabitants of the Northern Region of Malawi have limited but increasing access to cellular phones and data networks [11].

The percentage of the world’s population with access to mobile phones continues to increase. A 2014 study by the Pew Research Center found that 82% of Kenyans, 73% of Tanzanians, and 65% of Ugandans have a cell phone (compared to a rate in the United States of 89%) [12]. Increasing market coverage throughout sub-Saharan Africa, coupled with the ever-more affordable options for mobile phone ownership have resulted in growing research interest in the epidemiologic potential of mobile health. Despite this potential for mobile health surveillance, data on mobile phone based epidemiology remains scarce [13].

## METHODS

A cross-sectional mobile health survey focusing on maternal health was carried out between October and November 2017. The survey consisted of 4 eligibility questions concerning the respondent’s age, whether or not she had recently given birth, whether a death had occurred following the birth, and whether the respondent was answering for a household member who had died after childbirth. Eligible respondents then answered 36 questions about their prenatal services, birth attendants, location of delivery, and demographic data. Participants were excluded if they had not given birth in the last 12 months.

Survey questions were designed by the study authors and underwent multiple revisions. The content of the survey was evaluated by medical faculty both at the University of Southern California and at the Chitipa District Health Office (DHO). Questions regarding socioeconomic status were modeled on questions used by the DHS for surveys conducted in the region. The survey was further revised and edited for cultural variations in language (eg, ultrasound is referred to as “scanning” in the local spoken English) based upon feedback from the volunteers elicited during training sessions.

Volunteer members of the community were selected by a representative from the Chitipa DHO. These volunteer community informants could speak, read, and write English fluently. They were assigned to a geographic catchment area near their residence where they would be responsible for conducting the surveys. They were instructed to complete one survey for each birth that had occurred within their catchment area either in the last 12 months or during the period when survey collection was active. The survey was conducted by the community informant in the language most appropriate to their community and was recorded in English. Many of these volunteers had experience working with the United Nations Children’s Fund (UNICEF) Health Surveillance Assistant program and thus had received training appropriate for community health interventions and basic preventative care. They were familiar with the health practices of their communities and recognized by their peers as knowledgeable in basic health care.

The survey was loaded onto touchscreen smartphones capable of running the app version of the Qualtrics survey platform. Community informants were given one day of instruction, during which time they received training on how to use the cell phone and survey platform, an explanation of all survey questions, and the opportunity to practice taking a survey. Three translators familiar with local linguistic variation were present during both training days to clarify questions regarding the adaptation of survey questions written in English into local languages. The community informants were allowed to keep the cell phone for the duration of the survey period. Uploads of completed survey data were performed periodically by a study author using a mobile WiFi router, at which time questions and technical issues posed by the community informants were also addressed.

The survey was conducted at two sites: the villages surrounding Nthalire and Misuku in the Chitipa District in the Northern Region of Malawi. One full day of training, follow-ups for data upload, and a full day of study audits performed after the survey collection period were repeated at both the Nthalire and Misuku sites.

All data analysis was performed using Qualtrics software (Qualtrics, Provo, Utah, USA). No surveys were excluded from analysis. The study design and survey were reviewed by the University of Southern California Institutional Review Board and determined to be exempt.

## RESULTS

A total of 915 survey responses were recorded by 21 community informants. Responses to every question were not required to complete the survey. These surveys recorded 621 live births and 4 cases of maternal mortality. This represents a maternal mortality rate of 0.64% (95% CI = 0.2% to 1.6%), roughly equal to the UNICEF estimate from 2015 of 634 per 100 000 live births, or 0.63% [14]. Age was recorded in 673 of the survey responses, and community informants recorded 120 births by adolescent mothers aged 15-19. This represents 17.8% (95% CI = 15.1% to 20.9%) of all recorded births, slightly higher than the UNICEF estimate of 143 per 1000 live births (14.3%) [14]. **Table 1** compares selected values captured by the survey to corresponding accepted values that appear in the UNICEF Maternal and Newborn Health Disparities 2015 report as well as the 2010 Malawi DHS. While many of these values are similar, the population surveyed in this particularly remote area of the Northern Region still vary from aggregate data in the DHS, as demonstrated in **Table 2** comparing water supply and housing materials to those living in remote locations surveyed by the DHS.

**Table 1:** Comparison between survey data and accepted comparable values

	Survey values (95% CI)	Accepted comparable value	Source
Overall mortality	0.6% (0.2% to 1.6%)	0.63%	[14]
Maternal age 15-19	17.8% (15.1% to 20.9%)	14.30%	[14]
Causes of mortality	Hemorrhage 3 of 4 respondents; Hemorrhage with Infection 1 of 4 respondents	Leading causes of maternal mortality 1. Hemorrhage, 2. Infection	[15]
Cesarean Sections	6.0% (4.3% to 8.1%)	5.4% (Northern Region)	[14]
Skilled Attendant at Birth	93.6% (91.3% to 95.2%)	91.6% (Northern Region)	[14]
Institutional delivery	94.9% (92.8% to 96.4%)	91.8% (Northern Region)	[14]
HIV prevalence	1.9% (1.0% to 3.3%)	5.1% (Northern Region)	[16]
Mean number of children ever born	2.9 (2.74 to 3.04)	2.79	[16]
Mean number of children living	2.8 (2.63 to 2.92)	2.48	[16]

CI – confidence interval

**Table 2:** Comparison between surveyed population and DHS population as demonstrated by differences in access to drinking water and home flooring materials*

	Percent of data	95% confidence interval	DHS total percent distribution	DHS percent distribution – urban	DHS percent distribution – rural
What is the household's source of drinking water?					
Tube well or borehole	35.8%	32.4% to 39.4%	62%	10%	72%
Unimproved water source (unprotected spring, well, or other surface water)	31.5%	28.2% to 35.0%	13%	2%	15%
Public tap/standpipe	13.4%	11.1% to 16.1%	10%	33%	6%
Protected dug well	13.1%	10.8% to 15.8%	4%	2%	4%
Piped water into dwelling/yard/plot	3.6%	2.5% to 5.3%	8%	41%	2%
Piped to neighbor	2.5%	1.6% to 3.9%	3%	12%	1%
Bottled water or other improved source	0.1%	0.0% to 0.8%	N/A	N/A	N/A
**What is the main material of the floor of your home?**					
Natural (earth/sand)	75.8%	72.5% to 78.8%	74%	29%	83%
Finished floor (tile/cement/carpet)	24.2%	21.2% to 27.5%	25%	71%	17%

*DHS – Demographic and Health Survey [16].

In addition to accurately collecting basic demographic data, the mobile health survey also proved capable of producing more nuanced data regarding health care access. In questions concerning preventative care for pregnant women, 51.7% of women were recorded as attending 4 antenatal care visits (95% CI: 47.8% to 55.7%), consistent with the 2015-2016 DHS survey value of 51% [16].

A question was included in the survey requesting the name of the health care center where women underwent cesarean section. This question served as a quality control measure, as the options included two locations where these procedures are regularly performed (Chitipa District Hospital and Mzuzu Central Hospital) as well as two health care centers that are not equipped to provide this service. 28 responses indicating cesarean section were recorded, all of which correctly provided the name of a center where these services are regularly performed.

Audits of the survey were comprised of qualitative responses by ten community informants and survey participants at each of the study locations. Audits were conducted by one of the study authors and involved a second administration of the survey followed by scripted questions. The script included both interview questions for the original respondent of the survey and the volunteer who administered the survey. The survey instrument and qualitative questions are included in Appendix S1 in the **Online Supplementary Document**. No discrepancies between the information recorded in the survey and the information when the survey was repeated during the audit were noted. The community informants were invited to comment on the survey length, technical problems with the touchscreen mobile phones, and whether or not there were survey questions that respondents declined to answer. Three cases of technical problems with mobile phones were reported, however all the polled community informants and respondents reported that the survey was appropriate in length and contained questions the respondents were comfortable answering. The survey platform utilized pre-existing technology from Qualtrics and was determined to be appropriately user-friendly and no issues were reported. The use of touchscreen devices was novel for majority of study volunteers, so the onsite training was absolutely necessary in ensuring they were comfortable using their devices.

The total out-of-pocket cost of the project, including but not limited to travel for investigators from the United States, ground travel in Malawi, and the cost for mobile phones, accessories, and cellular data access was approximately US$10 200, resulting in a cost of approximately US$ 11.14 per survey. Of the total cost, approximately 80% was spent on travel and lodging for investigators, while the costs for mobile device activation, WiFi, phone accessories, and training day logistics accounted for the remaining portion of the study budget.

## DISCUSSION

Understanding the epidemiology of extremely rural or impoverished populations remains one of the great challenges of global epidemiology and population health. Large scale efforts like the Demographic Health Survey (DHS) are expensive and may take months to complete, meaning that the lag between information collection and dissemination can be significant [17]. While the majority of surveys are moving away from paper collection toward mobile platforms and the DHS may also use community members to collect data, this study design represents a novel approach to population surveillance in developing communities. This effort presents a scalable solution that could be implemented to perform either continuous surveillance of health concerns like infectious disease or could be adjusted and implemented to capture specialized data in real-time. Community members could act as volunteers or as compensated employees who could be enlisted to help collect data points tailored on a minute-to-minute basis to meet the needs of local, national, or global health care organizations.

Large-scale surveys like the DHS are generally based on surveying dwellings and thus are impractical for unstable populations such as refugees or those experiencing homelessness. In these situations, the use of mobile health and strategically placed community informants with specific knowledge of local geography and migration could not only help to provide early warning of infectious disease in these vulnerable populations but could also play a role in the planning of future aid projects.

Another potential usage of community based data collection is the ability to crowdsource information about local health care needs, cultural beliefs, and current workflows before implementing aid-based interventions. It is estimated that many of the supplies sent as foreign aid go unused or are underutilized [18]. This is likely related to the fact that the medical systems and health care centers in different environments have distinct and specific needs that can be difficult to predict. Furthermore, even interventions that seem appropriate may not fit into the cultural context of a community and may therefore be underutilized. Previously described examples of this phenomenon include neonatal incubation units which are donated and never used, or which may need to be retrofitted to function properly in a low-resource hospital [19,20]. The ability to use mobile health and descriptive surveys to enlist the involvement of the community before supplies are selected for donation could improve efficiency and utilization overall.

The results of this survey demonstrate that information collected by community members about their peers can produce valid information regarding health status and access to care. Furthermore, the differences between the rurality of this population, as evidenced by the number of individuals dependent on unimproved water sources (31.5%) compared to “Urban” (2%) or “Rural” (15%) categories of the DHS, demonstrates that this population is subject to environmental pressures more extreme than in many other parts of the country and may therefore have unique health care needs.

While many of the summary values produced by the survey are similar to published values, the survey captured an HIV prevalence of 1.9% (95% CI: 1.0% to 3.3%) which is lower than the 2010 DHS value for this region of 5.1%. This may represent a difference in this extremely rural subgroup but may also represent a failure of the survey to capture sensitive information that individuals may not be comfortable disclosing to a member of their community. It is plausible that this study design captured more accurate data for questions without cultural stigma and that having the community informant conduct the survey led to an underreporting of HIV positivity.

Access to WiFi and lack of equipment is often cited as a barrier to the use of mobile health for addressing global health disparity. While this is a valid concern and was a difficulty encountered by the study authors, the market for both cellular phones and wireless connectivity in the developing world is constantly growing and thus mobile health will become more practical and feasible to implement in coming years. At the time of the data collection for the study, WiFi was unavailable in the most remote study areas and uploads were completed in a nearby area by one of the study authors. For future projects a satellite internet modem or other portable connectivity device could provide the ability to collect data as surveys are completed.

A concern often raised in the context of mobile health and data collection is difficulty in maintaining participants interest in inputting data or participating in a survey. In the US, “survey fatigue” is often blamed for poor response rate, and new apps are so plentiful that only 25% of users will open an app for a second time after downloading [21]. The data from this study, however, demonstrates that community members will provide a sustained effort to input data. While limited access to WiFi connectivity presents challenges for data upload, it also means that respondents spend less time online and therefore are not subject to constant requests to complete surveys such as in the United States.

During the one-day training on phone and survey usage, the importance of inputting accurate data was explained to the community informants. They were also invited to suggest changes or additions to the survey before data collection began. The authors credit the successful data collection to emphasis on the importance of community members and their unique understanding of the local culture and epidemiology.

## CONCLUSION

Mobile health has the potential to provide efficient and timely data on the health of local populations. Using community members as reporters of health care data can provide accurate and reliable information even for populations in rural, low-resource, or unstable environments.

## Additional material

Online Supplementary Document
