# Supplementary material for: Utilizing mobile health and community informants to collect real-time health care data in extremely low resource environments
Source: J Glob Health. 2020 Nov 8;10(2):020411. doi: 10.7189/jogh.10.020411 (PMC7688196; doi:10.7189/jogh.10.020411)
Supplement: Online Supplementary Document [file jogh-10-020411-s001.pdf]

Appendix S1.- Survey Instrument

Eligibility Questions

1. Did a woman in your household (living or deceased) have a baby in the last year?
    - Yes, I had a baby in the last year.
    - Yes, I'm answering for someone else.
    - No.

2. Did the woman who gave birth survive for 42 days after childbirth?
    - Yes
    - No

3. How old are you (or how old is the woman you are answering for)?

4. If she died, do you know the cause of death? (Select all that apply)
    - Hemorrhage
    - Infection/fever
    - Eclampsia (Convulsions)
    - Trauma
    - Other ____

Survey Questions

5. Before becoming pregnant, did you have any chronic conditions? Select all that apply.
    - Tuberculosis
    - BP (Hypertension)
    - Diabetes
    - Asthma
    - Other ____
    - None

6. Including your most recent pregnancy, how many times in your life have you been pregnant?

7. How many times have you given live birth?

8. How many living children do you have?

9. During your most recent pregnancy were you pregnant with twins?
- No, I was pregnant with one baby
- Yes, I was pregnant with twins

10. Where did you give birth when you delivered the child (or twins) born this year?
- Hospital or healthcare center
- Own home
- Home of TBA
- On the way to a hospital or healthcare center

11. Please provide the name of the facility where you gave birth
- Nthalire Health Center
- Chitipa District Hospital
- Misuku Health Center
- Mzuzu Central Hospital
- Other ____

12. Who assisted with labor and birth? Select all that apply.
- Doctor
- TBA
- Midwife/Nurse
- Guardian/Relative/Friend

13. Did a doctor or healthcare worker tell you that your baby was underweight, normal or overweight? If not, please estimate:
- Underweight (less than 2.5kg)
- Normal (2.5-3.9kg)
- Overweight (more than 4kg)
- Don't know

14. If the baby died, when did they die?
- Stillborn
- Within one week of birth
- Within one month of birth
- Within one year of birth
- Not applicable

15. If you delivered at the hospital, how long were you in the labor ward?

- 4 hours or less
- 4 to 8 hours
- 8 to 12 hours
- 12 hours or more
- Don't know

16. Did you experience any complications before, during, or after you gave birth? Select all that apply.

- Bleeding
- Eclampsia (Convulsions)
- Infections (Fever)
- Surgery
- No complications

17. How many antenatal checkups did you have?
18. Where did you receive antenatal care?

- Hospital or healthcare facility
- Village clinic

19. How did you get there? Select all that apply.

- Walk
- Bicycle
- Oxcart
- Motorbike
- Car or Bus
- Other ____

20. How long did you travel for antenatal care?

- less than 1 hour
- 1-2 hours
- 2-3 hours
- 3-4 hours
- 4-5 hours
- more than 5 hours

21. During antenatal care, which of the following services did you receive? Select all that apply.

- Check vital signs (blood pressure, weight, temperature, etc.)
- Fetal heart check
- Ultrasound (Scanning)
- Supplements
- Cervical cancer screening

22. Have you been tested for HIV?
    - Yes
    - No

23. When were you tested for HIV?

24. What is your HIV status?
    - Positive
    - Negative

25. If positive, are you on treatment?
    - Yes
    - No
26. Was the child (or twins) tested for HIV?
    - Yes
    - No

27. What is the child's HIV status?
    - Positive
    - Negative
    - I had twins

28. Is your child (or twins) being treated for HIV?
    - Yes
    - No
29. What was the mode of birth?
    - Vaginal delivery
    - Caesarean section
    - Instrumental delivery
30. Have you ever had a cesarean section?
    - Yes
    - No

31. If so how many?

32. Where was the procedure performed?
    - Chitipa District Hospital
    - Mzuzu Central Hospital
    - Nthalire Health Center
    - Misuku Health Center

33. What is the highest level of school you attended:
    - Did not attend
    - Preschool
    - Primary
    - Secondary
    - Tertiary
    - Don't know

34. What is the main material of the floor of your home?
    - Natural (earth/sand)
    - Finished floor (tile/cement/carpet)

35. What is the household's source of drinking water?
    - Unimproved water source (unprotected spring, well, or other surface water)
    - Protected dug well
    - Tube well or borehole
    - Public tap/standpipe
    - Piped to neighbor
    - Piped water into dwelling/yard/plot
    - Bottled water or other improved source

36. Does anyone in the household have one of the following items? Select all that apply.
    - Radio
    - Television
    - Mobile phone
    - Refrigerator
    - Bicycle
    - Oxcart
    - Maize Storage
    - Cattle
    - Goats
    - Pigs

Appendix B - Audit Questions

Section 1: Questions for the HSA

a. How difficult was it to find participants for the survey? Did you have any challenges?
b. What was the process of administering the survey like for you?
c. How do you feel about using mobile phones in this study?
d. If you were to rank your level of comfort using the mobile phones in this study from 1 - 10 (1 being not at all comfortable, and 10 being the most comfortable), what number would you be? Why?

e. How do you feel about the survey?
f. Do you have any other comments you would like to share?

Section 2: Questions for the Survey Participant

a. Was the survey conducted?
b. If yes, was the survey administered by the HSA here?
c. Approximately how long did it take to complete the survey?
d. How do you feel about the length of the survey?
e. How do you feel about the survey?
f. (with the HSA out of the room) Did you feel that your survey was conducted in an adequately private area?
g. (with the HSA out of the room) Did you feel comfortable answering all the questions?
h. (with the HSA out of the room) Did you feel comfortable giving this information to the HSA?
i. Was there anything you think we should have asked that we didn't?
j. Were there any questions you didn't understand? (show participant the survey questions) Did the HSA take the time to explain them to you?
k. Do you have any other comments you would like to share?
